# Telehealth During COVID-19: Suicide Prevention and American Indian Communities in Montana

**DOI:** 10.1089/tmj.2021.0104

**Published:** 2022-03-10

**Authors:** Zachary Pruitt, Kate P. Chapin, Haley Eakin, Annie L. Glover

**Affiliations:** ^1^ College of Public Health, University of South Florida, Tampa, Florida, USA.; ^2^ Center for Children, Families, and Workforce Development, University of Montana Missoula, Missoula, Montana, USA.

**Keywords:** telehealth, vulnerable populations, telemedicine, suicide prevention

## Abstract

**Background:**

Public health measures that prevent the spread of COVID-19, such as social distancing, may increase the risk for suicide among American Indians due to decreased social connectedness that is crucial to wellbeing. Telehealth represents a potential solution, but barriers to effective suicide prevention may exist.

**Materials and Methods:**

In collaboration with Tribal and Urban Indian Health Center providers, this study measured suicide prevention practices during COVID-19. A 44-item Likert-type, web-based survey was distributed to Montana-based professionals who directly provide suicide prevention services to American Indians at risk for suicide. Descriptive statistics were calculated for survey items, and Mann–Whitney *U* tests examined the differences in telehealth use, training, skills among Montana geographic areas, and barriers between providers and their clients/patients.

**Results:**

Among the 80 respondents, two-thirds agreed or strongly agreed that American Indians experienced greater social disconnection since the COVID-19 pandemic began. Almost 98% agreed that telehealth was needed, and 93% were willing to use telehealth for suicide prevention services. Among current users, 75% agreed telehealth was effective for suicide prevention. Over one-third of respondents reported using telehealth for the first time during COVID-19 pandemic, and 30% use telehealth at least “usually” since the COVID-19 pandemic began, up from 6.3%. Compared with their own experiences, providers perceive their American Indian client/patients as experiencing greater barriers to telehealth.

**Discussion:**

Telehealth was increasingly utilized for suicide prevention during the COVID-19 pandemic. Opportunities to improve telehealth access should be explored, including investments in telehealth technologies for American Indians at risk for suicide.

## Introduction

Beyond the primary physical harms of COVID-19, the secondary consequences of the coronavirus pandemic, such as social isolation, economic stress, and barriers to mental health treatment, may increase the risk of suicide.^1,2^ Montana's consistently high suicide rate is among the highest in the nation^3^ and major public health concern for American Indian people throughout the United States.^3,4^ Overall, American Indian and Alaska Native community members are at a higher risk of suicide than any other racial or ethnic group in the United States.^5^ Mental health experts who work with American Indian communities in Montana fear the pandemic could make their mental health worse.^6^ Therefore, suicide prevention services require special attention during the coronavirus pandemic.^7,8^

## Background

Public health measures designed to reduce the risk of transmission and exposure to the coronavirus, such as social distancing, quarantine, and isolation, make face-to-face health care visits impracticable.^9^ As a result, health providers have been compelled to adopt telehealth technologies.^10,11^ Telehealth is an umbrella term that includes mHealth, telemedicine, teletherapy, telemental health, telebehavioral health, and telepsychotherapy, and technology that includes telephone, e-mail, text, videoconferencing platforms, and chat applications.^12^

Although not statistically different than in-person mental health care, research has found that telehealth significantly reduces depression symptoms and overall psychological distress.^13,14^ However, telehealth research has uncovered provider challenges with software and equipment usability and costs, issues of privacy and security, uncertainty about reimbursement, and skepticism about the efficiency, effectiveness, privacy, and security of telehealth.^15–18^

Furthermore, barriers to telehealth exist.^19,20^ Those living in rural areas or on Tribal lands are less likely to have access to high-speed connection to the internet, a necessary condition for telehealth.^21,22^ Also, telehealth equipment, such as hardware and software, can be a barrier to telehealth.^18,23,24^

Growth in telehealth adoption was driven in part by rural health centers' need to improve access to behavioral health care, especially given challenges associated with patients traveling long distances to clinics and the need to solve the shortage of mental health providers.^18^ Yet, increased use in telehealth has been hindered by resistance from some care providers who have been unwilling to adopt such technological innovations.^19,23,25^ This telehealth resistance is higher among Baby Boomers (ages 56 to 73) who have been found to be less comfortable with digital technology, compared with Generation X (ages 40 to 55), Millennials (ages 24 to 39) and Generation Z providers (age 24 and under).^26^ However, since the coronavirus pandemic, providers may be increasingly open to using telehealth as an option for delivery of the various aspects of suicide prevention service, including identification of warning signs, treatment for those at elevated risk for suicide, and transition support for patients navigating the health system.^15^

This study sought to identify perceptions and attitudes of behavioral health and medical care providers on the impact of the coronavirus pandemic on suicide risks among American Indians in Montana, changes in suicide prevention care, adoption of telehealth, and the effectiveness and efficiency of telehealth for suicide prevention services.

## Methods

A web-based survey of behavioral health and medical care providers who interact with individuals who may be at risk for suicide was conducted during October and November of 2020. The authors collaborated with Tribal and Urban Indian Health Center providers to develop the 44-item, Likert-style, web-based survey. The survey was designed to investigate respondent perceptions on concepts related to changes in protective and risk factors of suicide during the coronavirus pandemic, suicide prevention practices during the pandemic, the effect of the pandemic on use of telehealth, telehealth training and skills, and telehealth privacy and security. The Institutional Review Board of the University of Montana approved the study under the exempt category of review in October 2020 (IRB#143–20). See Supplementary Appendix for the web-based survey instrument.

Respondents were recruited from a series of five emails distributed over a 5-week period to 222 Montana-based behavioral health and medical care professionals on listservs and targeted e-mail lists. Three separate social media posts were made to Facebook and Instagram to followers (2,000 and 800, respectively) during that period. Respondents included behavioral health providers, medical care providers, and nonclinical staff who work directly with clients/patients to prevent suicides among American Indian communities of Montana. Participants who did not directly interact with individuals who may be at risk for suicide were excluded. Participants who completed the anonymous survey had the option to sign up for a weekly raffle for a $30 gift card. The final convenience sample included 80 respondents.

In addition, the survey collected information on the respondents regarding gender; race, ethnicity, or origin; age group; whether they were a member of a Tribal nation; zip code; primary patient focus (children, adolescents, adults, older adults, all groups equally); and occupation. Age groups were categorized to match the generational cohorts: under 24 years (Generation Z), 24 to 39 (Millennials), 40 to 55 (Generation X), 56 to 73 (Boomers), and over 74 years (Silent).^22^ Zip codes were categorized into geographic areas based on Rural–Urban Commuting Area Codes classification (RUCA version 2.0), including metropolitan area (cities of 50,000 and greater population), near metropolitan (outside core metropolitan area, but within an area that experiences high commuting to metropolitan area), urban cluster (cities/towns of from 2,500 through 49,999 populations), small town (2,500 through 9,999 populations), and remote (60 min or greater one-way road travel to the closest edge of an Urban Cluster of 10,000 or more).^27^ Respondent occupational categories were based on the Zero Suicide Workforce Survey, including an “other” category for those who interact with individuals who may be at risk for suicide but did not identify with one of the 11 defined occupational categories.^28^

For the Likert-type survey items, responses for each response option were scored (strongly agree = 5, agree = 4, neutral = 3, disagree = 2, strongly disagree = 1), and the percentage and count of each response option were calculated. The Mann–Whitney *U* tests compared the metropolitan geographic categories (metro and near-metro) compared with nonmetro geographies (urban cluster, small town, and remote) for differences in agreement in, telehealth training received, telehealth skills required, internet speed, telehealth equipment, whether telehealth was needed during the pandemic, whether telehealth was effective for suicide prevention, and the frequency of telehealth use during the coronavirus pandemic.^29^ Also, Mann–Whitney *U* tests examined the differences in how respondents report their internet speed and equipment as barriers compared with how they perceived their American Indian clients' and patients' internet speed and equipment as barriers.

## Results

Among the 191 surveys initiated, 111 were excluded (3 respondents did not agree to the informed consent statement, 60 did not directly interact with individuals who may be at risk for suicide, and 48 did not complete the survey in its entirety). The final sample included the analysis of 80 respondents who directly interact with individuals who may be at risk for suicide, either in person or from a distance. No respondents over 74 years of age completed the survey.

*Table 1* shows the characteristics of the survey respondents. Almost two-thirds of the respondents reported living in the metro areas (57.5%), such as Billings and Missoula, or outside core metropolitan areas but within a commutable distance (6.3%). Fifteen percent were from small towns, such as Miles City and Livingston, and 10% were living on Indian Reservations, including Blackfeet, Crow, Flathead, and Fort Belknap. The rest of the providers (7.5%) were from urban clusters, including Bozeman, Butte, and Kalispell. Six respondents preferred not to answer with their zip code.

**Table 1.** Behavioral Health and Medical Care Provider Respondent Characteristics (*n* = 80)

CHARACTERISTIC	*n*	%
Gender of Respondents		
Female	60	75.0
Male	17	21.3
Two spirit, transgender, gender nonbinary, or other	1	1.3
Preferred not to answer	2	2.5
Age range (Generation)		
Under 24 years old (Gen Z)	10	12.5
24 to 39 years old (Millennial)	21	26.3
40–55 years old (Gen X)	30	37.5
56 to 73 years old (Boomer)	15	18.8
74 and older (Silent Generation)	0	0.0
Prefer not to answer	4	5.0
Race, Ethnicity, or Origin of Respondents		
White	58	72.5
American Indian or Alaska Native	11	13.8
Hispanic, Latino, or Spanish Origin	3	3.8
Both White and American Indian or Alaska Native	3	3.8
Asian	1	1.3
Some other race or origin	1	1.3
Preferred not to answer	3	3.8
Member of a Tribal Nation		
No	64	80.0
Yes (Blackfeet Tribe, Chippewa Cree Tribe, Fort Belknap Tribes, Fort Peck Tribes)	10	12.5
Not an enrolled member of a Tribal Nation, but familial association with one or more Tribal Nations	4	5.0
Preferred not to answer	2	2.5
Which of the following groups do you primarily work with?		
Children	6	7.5
Adolescents	9	11.3
Adults	30	37.5
All groups equally	35	43.8
Geographic category based on population		
Metropolitan area (50,000 and greater populations)	46	57.5
Near metropolitan (high commuting to metro area)	5	6.3
Urban cluster (2,500 through 49,999 populations)	6	7.5
Small town (2,500 through 9,999 populations)	12	15.0
Remote (60 min or greater one-way road travel to the closest edge of an urban cluster of 10,000 or more)	5	6.3
Prefer not to answer	6	7.5
Respondent occupation		
Behavioral health clinical worker (Substance Abuse Counselor, Therapist, Psychologist)	21	26.3
Nursing (Nurse, Registered Nurse)	12	15.0
Management (Administrators, Supervisors, Managers, Coordinators)	11	13.8
Physical Health Care/Medication Management (Physician, Nurse Practitioner, Physician's Assistant)	7	8.8
Psychiatry (Psychiatrist, Psychiatric Nurse Practitioner)	7	8.8
Case Management	4	5.0
Business, Administrative, and Clerical (Accounting, Reception, Human Resources, Billing, Records, Information Technology)	3	3.8
Support and Outreach (Outreach, Faith, Family Support, Peer Support)	3	3.8
Education (Teacher, Health Educator)	2	2.5
Crisis Services	1	1.3
Patient Observer	1	1.3
Technician (Mental Health Technician, Behavioral Technician, Patient Care Assistance, Residential Technician)	1	1.3
Other	7	8.8

Before the COVID-19 pandemic, over half of respondents (58%) never used telehealth for suicide prevention practices. Approximately 36% of respondents reported using telehealth for the first time during COVID-19 pandemic. Thirty percent of respondents use telehealth “usually” or “every time” for suicide prevention practices since the COVID-19 pandemic began, up from 6.3%. There were no statistical differences in telehealth use since the coronavirus pandemic began for respondents living in the metropolitan geographic categories (metro and near-metro) compared with those living in nonmetro geographies (urban cluster, small town, and remote) (z-score = 1.20; *p* = 0.23). Respondents who reported using multiple telehealth technologies indicated the use of videoconferencing platforms (24%), telephone (24%), email (19%), texting (13%), smartphone applications (13%), and social media platforms (7%).

*Table 2* reports the results of respondent agreement related to suicide protective and risk factors during COVID-19 pandemic. Large majorities of respondents agreed with statements regarding the increased risk of suicide associated with the COVID-19 pandemic, such as “more social disconnection” (66.3%), “less likely to engage in community event” (73.8%), and “less likely to seek suicide prevention services” (61.3%).

**Table 2.** Perceptions of the Coronavirus Pandemic and Telehealth Among Behavioral Health and Medical Care Providers in Montana (*n* = 80)

	STRONGLY AGREE		AGREE		NEUTRAL		DISAGREE		STRONGLY DISAGREE		MEAN
	*n*	%	*n*	%	*n*	%	*n*	%	*n*	%	
Perceptions of suicide-protective and risk factors among American Indian clients/patients during COVID-19 pandemic											
Due to the COVID-19 pandemic that began in March 2020, American Indian clients/patients feel more socially disconnected from other people.	25	31.3	28	35.0	13	16.3	14	17.5	0	0.0	3.80
Due to the COVID-19 pandemic that began in March 2020, I believe that the American Indian communities I work with are less likely to engage in community-based events.	25	31.3	34	42.5	14	17.5	6	7.5	1	1.3	3.95
The COVID-19 pandemic that began in March 2020 has made American Indian clients/patients less likely to seek suicide prevention services, such as using self-help resources or making appointments with health providers?	20	25.0	29	36.3	25	31.3	6	7.5	0	0.0	3.79
Changes in suicide prevention practices during COVID-19 pandemic among providers directly caring for American Indian clients/patients											
The COVID-19 pandemic that began in March 2020, has changed how I identify warning signs for suicide.	5	6.3	31	38.8	18	22.5	15	18.8	11	13.8	3.05
The COVID-19 pandemic that began in March 2020 has changed my ability to provide care to individuals who have been identified as being at elevated risk for suicide.	18	22.5	37	46.3	12	15.0	11	13.8	2	2.5	3.73
The COVID-19 pandemic that began in March 2020 has changed the skills I need to work with individuals during their transitions in care.	19	23.8	34	42.5	18	22.5	5	6.3	4	5.0	3.74
Effect of COVID-19 pandemic on use of telehealth for suicide prevention services provided to American Indian clients/patients											
Telehealth is needed to assure American Indian communities of Montana to have access to care to prevent suicide during the COVID-19 pandemic.	62	77.5	16	20.0	2	2.5	0	0.0	0	0.0	4.75
Telehealth is effective in preventing suicides among Montana's American Indian communities during the COVID-19 pandemic.	26	32.5	27	33.8	24	30.0	1	1.3	2	2.5	3.93
I am willing to use telehealth for American Indian communities of Montana to provide suicide prevention practices during the COVID-19 pandemic.	51	63.8	24	30.0	5	6.3	0	0.0	0	0.0	4.58
During a telehealth appointment, I am just as effective at recognizing when an individual may be at elevated risk for suicide.	10	12.5	23	28.8	18	22.5	11	13.8	1	1.3	2.74
During a telehealth appointment, I am just as effective at responding when I suspect an individual may be at elevated risk for suicide.	11	13.8	34	42.5	13	16.3	4	5.0	1	1.3	2.99
During a telehealth appointment, I am just as effective at asking individuals direct and open questions about suicidal thoughts and behaviors.	23	28.8	29	36.3	7	8.8	3	3.8	1	1.3	3.24
During a telehealth appointment, I am just as effective at providing treatment to individuals with suicidal thoughts or behaviors.	11	13.8	28	35.0	16	20.0	7	8.8	1	1.3	2.88
During a telehealth appointment, I am just as effective at working with individuals during their transitions in care.	7	8.8	16	20.0	27	33.8	12	15.0	1	1.3	2.56
I think telehealth is more time consuming than face-to-face visits.	3	3.8	13	16.3	30	37.5	27	33.8	7	8.8	2.73
Telehealth training and skills/capabilities among those who provide suicide prevention services to American Indian clients/patients											
I have received training related to providing telehealth services.	13	16.3	26	32.5	14	17.5	19	23.8	8	10.0	3.21
I have the technology-related skills and capabilities to provide telehealth services.	20	25.0	41	51.3	13	16.3	5	6.3	1	1.3	3.93
Perceptions of telehealth privacy and security among those who provide suicide prevention services to American Indian clients/patients											
I think telehealth assures the privacy for American Indian communities of Montana who are at risk for suicide.	17	21.3	24	30.0	32	40.0	6	7.5	1	1.3	3.63
I think telehealth is secure for American Indian communities of Montana who are at risk for suicide.	18	22.5	25	31.3	33	41.3	3	3.8	1	1.3	3.70

When responding to the changes of suicide prevention practices since COVID-19 pandemic began, respondents strongly agree or agree that services have changed during the COVID-19 pandemic, such as providing suicide prevention treatment (68.8%) and supporting transitions in care (66.3%). The agreement is not as strong that the pandemic changed how providers identify warning signs, although 45% strongly agree or agree. A large majority (93.8%) reported agreement to the statement “I am willing to use telehealth for American Indian communities of Montana to provide suicide prevention practices during the COVID-19 pandemic.” Only a small proportion (20%) reported telehealth was more time consuming than face-to-face visits. However, according to respondents, 30% were never able to bill to insurance for suicide prevention practices provided through telehealth. Finally, over half of respondents strongly agreed or agreed that telehealth protected privacy (51.3%) and security (53.8%), a substantial minority were neutral about whether telehealth protected privacy (40.0%) and security (41.3%), and less than 10% on each item strongly disagree or disagreed.

*Table 2* shows the perceived effect of the COVID-19 pandemic on respondents' use of telehealth. Approximately two-thirds (66.3%) of respondents strongly agreed or agreed that telehealth is effective in preventing suicides among Montana's American Indian communities during the COVID-19 pandemic. Closer analysis shows that the proportion in agreement (strongly agree and agree) regarding telehealth being effective for suicide prevention increases to approximately three-quarters (74.6%) among those who reported using telehealth. When examining generational cohorts of respondents, the level of agreement (strongly agree and agree) about whether telehealth is effective decreases as the age groups increase. That is, among the 76 individuals who reported their age group, Gen Z had the highest proportion of agreement that telehealth is effective (*n* = 10, 80%), followed by Millennials (*n* = 21, 76%), Gen X (*n* = 30, 63%), and Baby Boomers (*n* = 15, 47%).

*Table 2* also reports the results of the effectiveness of telehealth for specific types of suicide prevention practices. Respondents strongly agree or agree that they are “just as effective” at recognizing when an individual may be at elevated risk for suicide (41.3%), responding when an individual may be at elevated risk for suicide (56.3%), asking individuals direct and open questions about suicidal thoughts and behaviors (65.0%), and providing treatment to individuals with suicidal thoughts or behaviors (48.8%). Respondents reported the least agreement (28.8%) that they are effective at working with individuals during their transitions in care.

Almost half of respondents (48.8%) reported having received training related to providing telehealth services. When examined by geographic area, *Table 3* shows the levels of agreement among providers regarding items related to telehealth training and skills/capabilities. When categorized as “metro” (metro and near-metro combined) and “nonmetro” (urban cluster, small town, and remote, combined), there were no statistical differences in the telehealth training item by metropolitan geographic categories, compared with nonmetro geographies (z-score = −1.16; *p* = 0.25). Also, there were no statistical differences regarding agreement about technology-related skills and capabilities to provide telehealth services between “metro” and “nonmetro” respondents (*p* = 0.65). There were also no statistical differences in internet speed (z-score = 1.27; *p* = 0.20) or telehealth equipment (z-score = −0.54; *p* = 0.59) between respondents of “metro” and “nonmetro” geographical areas.

**Table 3.** Provider Agreement Training and Skills/Capabilities by Rural–Urban Commuting Area Codes Geographic Areas (*n* = 76)

ROW LABELS	STRONGLY AGREE		AGREE		NEUTRAL		DISAGREE		STRONGLY DISAGREE		TOTAL
	*n*	%	*n*	%	*n*	%	*n*	%	*n*	%	*n*
Telehealth training											
Metropolitan area (*n* = 46)	5	10.9	14	30.4	6	13	15	32.6	6	13.0	46
Near metropolitan (*n* = 5)	2	40.0	3	60.0	0	0	0	0.0	0	0.0	5
Urban cluster (*n* = 6)	1	16.7	1	16.7	2	33	1	16.7	1	16.7	6
Small town (*n* = 12)	3	25.0	4	33.3	4	33	1	8.3	0	0.0	12
Remote (*n* = 5)	1	20.0	1	20.0	2	40	1	20.0	0	0.0	5
Telehealth skill/capabilities											
Metropolitan area (*n* = 46)	10	21.7	25	54.3	6	13.0	4	8.7	1	2.2	46
Near metropolitan (*n* = 5)	2	40.0	3	60.0	0	0.0	0	0.0	0	0.0	5
Urban cluster (*n* = 6)	1	16.7	2	33.3	2	33.3	1	16.7	0	0.0	6
Small town (*n* = 12)	3	25.0	6	50.0	3	25.0	0	0.0	0	0.0	12
Remote (*n* = 5)	2	40.0	1	20.0	2	40.0	0	0.0	0	0.0	5

*Table 4* shows results regarding the perceptions of barriers to telehealth. The survey asked respondents to assess barriers to effective telehealth, including their internet speed and telehealth equipment. Respondents were also asked to assess the same barriers for their American Indian clients or patients. Few respondents perceived that their internet speed (6.3%) or equipment (6.3%) were serious barriers to telehealth. However, about one-third of the respondents reported that their American Indian clients/patients had serious barriers for internet speed (31.3%) and telehealth equipment (36.3%). When comparing the items statistically, there were significant differences between how respondents report their internet speed and equipment as barriers compared with how they perceived their American Indian clients' and patients' internet speed (*p* < 0.001) and equipment as barriers (*p* < 0.001), respectively.

**Table 4.** Perceptions of Barriers to Telehealth Among Behavioral Health and Medical Care Provider Respondents Providing Suicide Prevention (*n* = 80)

	NOT AT ALL A BARRIER		MINOR BARRIER		MODERATE BARRIER		SERIOUS BARRIER		z-SCORE^a^
	*n*	%	*n*	%	*n*	%	*n*	%	
My internet speed is a barrier to using telehealth for suicide prevention practices for Montana's American Indian communities.	32	40.0	31	38.8	12	15.0	5	6.3	−6.27^***^
The internet speed for Montana's American Indian communities is a barrier to using telehealth for suicide prevention practices.	5	6.3	20	25.0	30	37.5	25	31.3	
My equipment is a barrier to providing effective telehealth for American Indian individuals at risk for suicide.	40	50.0	23	28.8	12	15.0	5	6.3	−6.74^***^
My American Indian clients'/patients' equipment is a barrier to providing effective telehealth for suicide prevention practices.	6	7.5	17	21.3	28	35.0	29	36.3	

^a^ The Mann–Whitney *U* tests calculated z-scores.

^***^ <0.001.

## Discussion

This survey indicates that Montana-based behavioral health and medical care providers perceive American Indian communities to have increased risk factors for suicide since the coronavirus pandemic began, as measured by perceptions of more social disconnection, fewer community events, and reduced access to suicide prevention services. These pandemic conditions are particularly problematic for behavioral health and medical care providers for whom best practices dictate that they focus on the strengths of American Indian communities, including connectedness with family, spirituality, environment, and community, as an approach to reduce suicide risk.^30–37^ In response to this increased risk, this study found that respondents are willing to provide suicide prevention services through telehealth, consistent with other studies regarding telehealth during the pandemic.^11,15^ Older generations are just as willing to use telehealth during the pandemic, contrary to the resistance sometimes found among Baby Boomers.^26^

Among those surveyed who use telehealth, most reported that they thought telehealth was effective in preventing suicides among American Indian communities in Montana during the COVID-19 pandemic. This survey also found that providers perceive suicide prevention services through telehealth as effective as face-to-face care (e.g., asking individuals direct and open questions and responding to those at risk for suicide), whereas providers viewed other tasks more ambivalently (e.g., transitions in care). This variation on the relative effectiveness of telehealth compared with face-to-face care delivery has been reported in other studies.^18,19,38^

Furthermore, the survey results demonstrated providers' varied perceptions of the efficiency of telehealth compared with face-to-face visits, consistent with recent research regarding telehealth during the pandemic.^15^ While the pandemic changed how respondents provided suicide prevention services, a large majority of subjects disagree, or remain undecided, as to whether “telehealth is more time consuming than face-to-face visits.”^15^

Earlier research highlights provider challenges with internet speed and telehealth equipment usability and costs.^16,19^ Although Lin et al. found that internet speed was not a barrier to telehealth for providers in rural health centers, our results of Montana-based providers show that over 75% perceive internet speed and equipment as not at all a barrier or a minor barrier to telehealth.^18^ Also, few respondents reported concerns about privacy and security of telehealth, a barrier to telehealth identified and expressed in other research.^19^

However, almost a third of respondents reported that they were never able to bill to insurance for suicide prevention practices provided through telehealth, echoing an uncertainty about telehealth reimbursement reported in other research.^11,19^ While payment and legal structures have evolved in response to the pandemic, telehealth payments have not yet achieved parity with face-to-face visits.^39,40^

This study hypothesized that RUCA-categorized metropolitan and near-metropolitan area providers' perceptions of telehealth use during the pandemic, telehealth training, telehealth skills, internet speed, and telehealth equipment would be different from those in nonmetro areas (urban cluster, small town, and remote). However, the statistical analysis failed to find differences in provider responses by geography, differing from Lin et al. who found that community health centers in rural locations were more likely to use telehealth.^18^

However, this survey shows significant disparities in telehealth access between providers and the American Indian communities they serve, limiting the potential to prevent suicides during the pandemic and underscoring the need to address telehealth technologies as an important public health issue.^21^ Investments in broadband internet access and telehealth equipment for at-risk American Indians could improve access to needed care and prevent suicides, even after the pandemic ends.^10^

The generalizability of the study should be carefully considered under the light of several limitations, most importantly the selection bias associated with the convenience sampling methodology. In addition, the small sample size and the low response rate (<36%) may limit the generalizability of the results. Although true of most surveys, the possible nonresponse error may influence the study results. Also, these results represent the first application of the web-based survey instrument, so no established reliability statistics confirmed that the questions would evoke consistent responses. Finally, the survey did not address client/patient perspectives on telehealth during the pandemic, so evidence about the relative difference in barriers between the providers and clients/patients is from the providers' perspectives only.

## Conclusion

According to these survey results, suicide risk among American Indian communities may have increased since the coronavirus pandemic began. Fortunately, the surveyed behavioral health and medical care providers reported increasing use of telehealth for suicide prevention care. Many providers used telehealth for the first time during the coronavirus pandemic, and most report a willingness to adapt to how they care for American Indians at risk for suicide.

The survey highlights opportunities to improve access to suicide prevention services by addressing perceived disparities in internet speed and telehealth equipment among American Indian communities. Investments in broadband internet access and telehealth equipment for American Indians at risk for suicide could improve access to needed care. Nevertheless, any suicide prevention program for American Indian communities, including investments in telehealth technologies, should use a strength-based approach to create culturally competent programs.^34^ Any program aimed at increasing telehealth use for American Indian communities should first assess the perceptions of telehealth, technology barriers, and the overall needs and preferences among American Indian communities.

In addition, telehealth training on certain aspects of suicide prevention, such as recognizing when an individual may be at elevated risk for suicide and supporting transitions of care through the health system, could make suicide prevention services more effective. Also, targeted training for older generations of medical care and behavioral health providers, who may be skeptical of the effectiveness of telehealth, may improve quality and access to care for suicide prevention services.

Continued expansion of telehealth for suicide prevention—viewed by respondents as needed during the pandemic, comparably time-efficient as face-to-face visits, and mostly effective for suicide prevention practices—could be an opportunity to improve access for American Indians in rural or Tribal lands where travel distance and a shortage of behavioral health providers will continue to be access to care barriers, even after the pandemic ends.

## Supplementary Material

Supplemental data
