# Supplementary material for: Telehealth During COVID-19: Suicide Prevention and American Indian Communities in Montana
Source: Telemed J E Health. 2022 Mar 10;28(3):325–33. doi: 10.1089/tmj.2021.0104 (PMC8968828; doi:10.1089/tmj.2021.0104)
Supplement: Supplemental data [file Supp_AppS1.docx]

**Supplementary** **Appendix: Provider Survey**

This survey measures perceptions, attitudes, and beliefs of behavioral health and medical providers who are involved in preventing suicides among American Indian communities of Montana during the COVID-19 pandemic.

1. As part of your role, do you directly interact with individuals (either in person or from a distance) who may be at risk for suicide? This includes things such as answering phones, scheduling appointments, conducting check-ins, and providing caregiving and/or clinical services.

- Yes
- No

**Section 1. Suicide Prevention Practices**

This section is intended to assess the extent to which your suicide prevention practices for American Indian communities of Montana have changed due to the COVID-19 pandemic. Please read each of the following statements about your attitudes, experiences, beliefs about your suicide prevention practices.

**(All: Strongly Disagree; Disagree; Neutral; Agree; Strongly Agree)**

1. The COVID-19 pandemic that began in March 2020, has changed how I identify warning signs for suicide.
2. The COVID-19 pandemic that began in March 2020 has changed my ability to provide care to individuals who have been identified as being at elevated risk for suicide.
3. Transitions in care are when clients/patients move from inpatient, emergency department, or primary care to outpatient behavioral health care. Examples include warm hand-offs or actions that assure clients/patients make their follow-up appointments. The COVID-19 pandemic that began in March 2020 has changed the skills I need to work with individuals during their transitions in care.

**Section 2. Suicide Protective Factors and Risk Factors**

This section is intended to assess the extent to which you perceive that suicide protective factors and risk factors have changed due to the COVID-19 pandemic. Please read each of the following statements about your perception of suicide risk factors during the COVID-19 pandemic.

**(All: Strongly Disagree; Disagree; Neutral; Agree; Strongly Agree)**

1. Due to the COVID-19 pandemic that began in March 2020, my American Indian clients/patients feel more socially disconnected from other people.
2. Due to the COVID-19 pandemic that began in March 2020, I believe that the American Indian communities I work with are less likely to engage in community-based events.
3. The COVID-19 pandemic that began in March 2020 has made American Indian clients/patients less likely to seek suicide prevention services, such as using self-help resources or making appointments with health providers?

**Section 3. Service Provision Practices for Suicide Prevention Workforce**

This section is intended to assess the extent to which you perceive the COVID-19 pandemic that began in March 2020 has changed clients’/patients’ access to effective suicide prevention practices. Suicide prevention practices include identification, screening, assessment, safety planning, treatment, transition care, and follow-up.

**(All: Strongly Disagree; Disagree; Neutral; Agree; Strongly Agree)**

1. Due to the COVID-19 pandemic that began in March 2020, the time spent with American my Indian clients/patients at each contact (i.e., length of visit) for suicide prevention care has increased.
2. Due to the COVID-19 pandemic that began in March 2020, the frequency of contact with my American Indian clients/patients for suicide prevention care has increased.

**Section 4. Frequency of Telehealth**

This section is intended to assess the frequency with which you use telehealth for suicide prevention practices for Montana’s American Indian clients/patients. Telehealth is an umbrella term including mHealth, telemedicine, teletherapy, telemental health, and telepsychotherapy. Telehealth includes telephone, email, text, videoconference, and other technology applications.

**(All of next three questions: Never; Rarely, in less than 10% of the chances when I could have; Occasionally, in about 30% of the chances when I could have; Sometimes, in about 50% of the chances when I could have; Frequently, in about 70% of the chances when I could have; Usually, in about 90% of the chances I could have; Every time)**

1. Prior to the COVID-19 pandemic that began in March 2020, I used telehealth for suicide prevention practices.
2. Since the COVID-19 pandemic began in March 2020, I use telehealth for suicide prevention practices.
3. Since the COVID-19 pandemic began in March 2020, I am able to bill to insurance for suicide prevention practices provided through telehealth, including identification, screening, assessment, safety planning, treatment, transition care, and follow-up.
4. [If not] Why are you not using telehealth for suicide prevention practices since the COVID-19 pandemic began in March 2020? (Check all that apply)

- No perceived need
- Organization resistant to change
- Lack of technological abilities among staff
- Cost of telehealth
- Lack of payment for telehealth
- Lack of high-speed internet access needed for telehealth
- Lack of software for telehealth
- Lack of equipment for telehealth
- Patient safety concerns
- Patient privacy concerns
- Provider licensing issues

**Section 5. Effect of COVID-19 Pandemic on Use of Telehealth**

This section is intended to assess your agreement regarding the use of telehealth for suicide prevention practices for Montana’s American Indian clients/patients. Telehealth is an umbrella term including mHealth, telemedicine, teletherapy, telemental health, and telepsychotherapy. Telehealth includes telephone, email, text, videoconference, and other technology applications.

**(All: Strongly Disagree; Disagree; Neutral; Agree; Strongly Agree)**

1. During a telehealth appointment, I am just as effective at recognizing when an individual may be at elevated risk for suicide.
2. During a telehealth appointment, I am just as effective at responding when I suspect an individual may be at elevated risk for suicide.
3. During a telehealth appointment, I am just as effective at asking individuals direct and open questions about suicidal thoughts and behaviors.
4. During a telehealth appointment, I am just as effective at providing treatment to individuals with suicidal thoughts or behaviors.
5. During a telehealth appointment, I am just as effective at working with individuals during their transitions in care.
6. Since the COVID-19 pandemic, which communication technology do you use to provide suicide prevention care remotely? (select all that apply)
   1. Email
   2. Smartphone app
   3. Social media platform
   4. Telephone
   5. Texting
   6. Videoconferencing platform

**(All: Strongly Disagree; Disagree; Neutral; Agree; Strongly Agree)**

1. Telehealth is needed to assure American Indian communities of Montana have access to care to prevent suicide during the COVID-19 pandemic.
2. Telehealth is effective in preventing suicides among American Indian communities of Montana during the COVID-19 pandemic.
3. I am willing to use telehealth for American Indian communities of Montana to provide suicide prevention practices during the COVID-19 pandemic.
4. I think telehealth is more time-consuming than face-to-face visits.
5. I think telehealth assures the privacy for American Indian communities of Montana who are at risk for suicide. Telehealth privacy risks involve a lack of control over the collection, use, and sharing of data.
6. I think telehealth is secure for American Indian communities of Montana who are at risk for suicide. Telehealth security risk involve unauthorized access to data through system flaws, such as hackers and malware.
7. I have the technology-related skills and capabilities to provide telehealth services.
8. I have received training related to providing telehealth services.

**Section 6. Service Provision Practices for Suicide Prevention Workforce**

This section is intended to assess the technology barriers to telehealth for suicide prevention practices among Montana’s American Indian communities.

**(All: Not at all a barrier; Minor barrier; Moderate barrier; Serious barrier)**

1. My internet speed is a barrier to using telehealth for suicide prevention practices for Montana’s American Indian communities.
2. The internet speed for American Indian communities of Montana is a barrier to using telehealth for suicide prevention practices.
3. My equipment is a barrier to providing effective telehealth for American Indian individuals at risk for suicide.
4. My American Indian clients’/patients’ equipment is a barrier to providing effective telehealth for suicide prevention practices.

**Section 7. You and Your Work Environment**

In this final section of questions, we would like to learn more about your work environment and your role within that environment.

1. Which best describes the location of where you most often work?

- Frontier (more than 60 minutes from a non-federal general hospital of 75 beds or more)
- Rural (less than 2,500 people)
- Urbanized cluster (at least 2,500 and less than 50,000 people)
- Urbanized area (at least 50,000 people)

1. Please choose the one category below that best describes your primary professional role.

- Adjunct Therapist (Activity, Occupational, Physical, Rehabilitation)
- Behavioral Health Clinician (Counselor, Social Worker, Substance Abuse Counselor, Therapist, Psychologist)
- Business, Administrative, and Clerical (Accounting, Reception, Human Resources, Billing, Records, Information Technology)
- Case Management
- Crisis Services
- Education (Teacher, Health Educator)
- Facility Operations (Dietary, Housekeeping, Maintenance, Security, Transportation)
- Management (Administrators, Supervisors, Managers, Coordinators)
- Nursing (Nurse, Registered Nurse)
- Patient Observer
- Physical Health Care/Medication Management (Physician, Nurse Practitioner, Physician’s Assistant)
- Psychiatry (Psychiatrist, Psychiatric Nurse Practitioner)
- Support and Outreach (Outreach, Faith, Family Support, Peer Support)
- Technician (Mental Health Technician, Behavioral Technician, Patient Care Assistance, Residential Technician)

1. In which of the following settings do you work?

- Inpatient setting
- Outpatient setting
- Both

1. You indicated that you directly interact with individuals either in person or from a distance who may be at risk for suicide during your day-to-day duties within the organization. Which of the following groups do you primarily work with?

- Children
- Adolescents
- Adults
- Elderly

1. Are you responsible for practicing suicide prevention services, including any of the following: identifying, screening, assessment, treatment, transition on care, or follow-up?

- Yes
- No

1. Which age range contains your current age?

- Under 24 years
- 24 to 39
- 40 to 55
- 56 to 73
- Over 74 years

1. What is your gender?

- Male
- Female
- Other or prefer not to say

1. What is your race or place of origin?

- White
- Hispanic, Latino, or Spanish Origin
- Black or African American
- Asian
- American Indian or Alaska Native
- Native Hawaiian or Other Pacific Islander
- Some other race or origin
- Prefer not to answer

1. Are you an enrolled member of a Tribal Nation?

- Yes
- No
- I am not an enrolled member of a Tribal Nation, but I have familial association with one or more Tribal Nations.

1. [If yes] In which Tribal Nation(s) are you enrolled?

- Blackfeet Tribe
- Chippewa Cree Tribe
- Confederated Salish & Kootenai Tribes
- Crow Tribe
- Fort Belknap Tribes
- Fort Peck Tribes
- Little Shell Chippewa Tribe
- Northern Cheyenne Tribe
- Other (space to fill in)

1. Do you currently live on an Indian Reservation?

- Yes
- No

1. [If yes] Please select the Indian Reservation where you currently live:

- Flathead Reservation
- Rocky Boy’s Reservation
- Fort Peck Reservation
- Fort Belknap Reservation
- Crow Reservation
- Northern Cheyenne Reservation
- Blackfeet Reservation
- Other (space to fill in)
